# Usability and Acceptance of the Embodied Conversational Agent Anne by People With Dementia and Their Caregivers: Exploratory Study in Home Environment Settings

**DOI:** 10.2196/25891

**Published:** 2021-06-19

**Authors:** Vera Stara, Benjamin Vera, Daniel Bolliger, Lorena Rossi, Elisa Felici, Mirko Di Rosa, Michiel de Jong, Susy Paolini

**Affiliations:** 1 Model of Care and New Technologies IRCCS INRCA - National Institute of Health and Science on Ageing Ancona Italy; 2 iHomeLab - University of Applied Sciences & Arts Lucerne Switzerland; 3 Unit of Geriatric Pharmacoepidemiology and Biostatistics IRCCS INRCA - National Institute of Health and Science on Ageing Ancona Italy; 4 Research Group IT Innovations in Healthcare Windesheim University of Applied Sciences Zwolle Netherlands; 5 Unit of Neurology IRCCS INRCA - National Institute of Health and Science on Ageing Ancona Italy

**Keywords:** dementia, older adults with dementia, embodied conversational agent, virtual personal assistant, virtual agent, virtual companion, design for older adults with dementia

## Abstract

**Background:**

Information and communication technologies are tools that are able to support cognitive functions, monitor health and movements, provide reminders to maintain residual memory abilities, and promote social support, especially among patients with dementia. Among these technologies, embodied conversational agents (ECAs) are seen as screen-based entities designed to stimulate human face-to-face conversation skills, allowing for natural human-machine interaction. Unfortunately, the evidence that such agents deliver care benefits in supporting people affected by dementia and their caregivers has not yet been well studied. Therefore, research in this area is essential for the entire scientific community.

**Objective:**

This study aims to evaluate the usability and acceptability of the virtual agent Anne by people living with dementia. The study is also designed to assess the ability of target users to use the system independently and receive valuable information from it.

**Methods:**

We conducted a 4-week trial that involved 20 older adults living with dementia and 14 family caregivers in home environment settings in Italy. This study used a mixed methods approach, balancing quantitative and qualitative instruments to gather data from users. Telemetry data were also collected.

**Results:**

Older users were particularly engaged in providing significant responses and participating in system improvements. Some of them clearly discussed how technical problems related to speech recognition had a negative impact on the intention to use, adaptiveness, usefulness, and trust. Moreover, the usability of the system achieved an encouraging score, and half of the sample recognized a role of the agent Anne. This study confirms that the quality of automatic speech recognition and synthesis is still a technical issue and has room for improvement, whereas the touch screen modality is almost stable and positively used by patients with dementia.

**Conclusions:**

This study demonstrated the ability of target users to use the system independently in their home environment; overall, the involved participants shared good engagement with the system, approaching the virtual agents as a companion able to support memory and enjoyment needs. Therefore, this research provides data that sustain the use of ECAs as future eHealth systems that are able to address the basic and higher-level needs of people living with dementia. This specific field of research is novel and poorly discussed in the scientific community. This could be because of its novelty, yet there is an urgent need to strengthen data, research, and innovation to accelerate the implementation of ECAs as a future method to offer nonpharmacological support to community-dwelling people with dementia.

## Introduction

### Background

The aging population around the world is growing rapidly, and dementia, as an age-dependent condition [1], has become a significant threat to global health. Worldwide, dementia could affect 47 to 132 million people by 2050, causing high impacts on individuals, families, communities, governments, and societies [2].

In this scenario, information and communication technologies, especially touch screens, are seen as tools that can support cognitive functions, monitor health and movements, provide reminders to maintain residual memory abilities, promote social support, improve communication with caregivers, and provide useful information concerning health conditions [3].

Among these technologies, embodied conversational agents (ECAs) or personal virtual assistants are seen as screen-based entities designed to stimulate human face-to-face conversational skills and thus allowing for natural human-machine interaction [4,5]. Unfortunately, the evidence that such agents are appropriate to deliver care benefits for supporting people living with dementia and their caregivers is yet to be studied. Therefore, research in this area is essential for the entire scientific community [6]. In fact, in the last 10 years, few studies have addressed the use of ECAs among older adults with dementia.

These include a tool for real-time streaming to a television of a realistic female avatar, previously programmed by a caregiver [7]. This avatar has a realistic voice, and the lips are in synchronization with its speech to ensure that its facial movements appear natural when reminders, notifications, and short dialogs with the user are used to support patients with dementia in their daily activities. In another study, a conversational agent system was shown on a computer screen in the form of an animated face resembling *a 5-year-old grandchild* [8]. This system can detect the end of the speech sound of a subject’s response to a question and ask the next question. When the subject speaks, the agent reacts by automatically generating nods, mouth movements, and acknowledgments. In this specific study, the ECA is seen as an alternative way of conversing when no human conversation partner exists. The animation of a female cartoon-like character was used to develop LOUISE [9-11], displayed in an idle pose and moving its lips while speaking on either a computer screen or a television set. This ECA includes attention monitoring and interaction management to automatically determine whether a person wants to communicate. Lastly, a humanoid female character was used to investigate different affective identities found in older care home residents with Alzheimer disease [12]. The challenges of involving patients with dementia, as well as the possibility of engaging them in a home environment for a significant period, are reported in the studies as common limitations and problems.

All the examples mentioned above prove that the ECA research area in the eHealth sector is still immature [13], but this newness can open up opportunities for the future, especially for the challenge of enabling people with dementia and their caregivers to better manage their lives. To bridge this gap, this paper discusses the main findings emerging after 4 weeks in which 20 older adults with dementia and 14 family caregivers used the ECA *Anne* in home environment settings in Italy.

### Objectives

This study aims to evaluate the usability and acceptability of the ECA Anne by older adults living with dementia. The study is also designed to assess the ability of target users to use the system independently and receive valuable information from it.

## Methods

### Overview

First, Anne was developed along the MyLifeMyWay project to enable seniors to live at home independently for as long as possible. The system was then adapted for seniors with forgetfulness, as is typical at the beginning of dementia, during the Living Well With Anne project.

**Figure 1:**
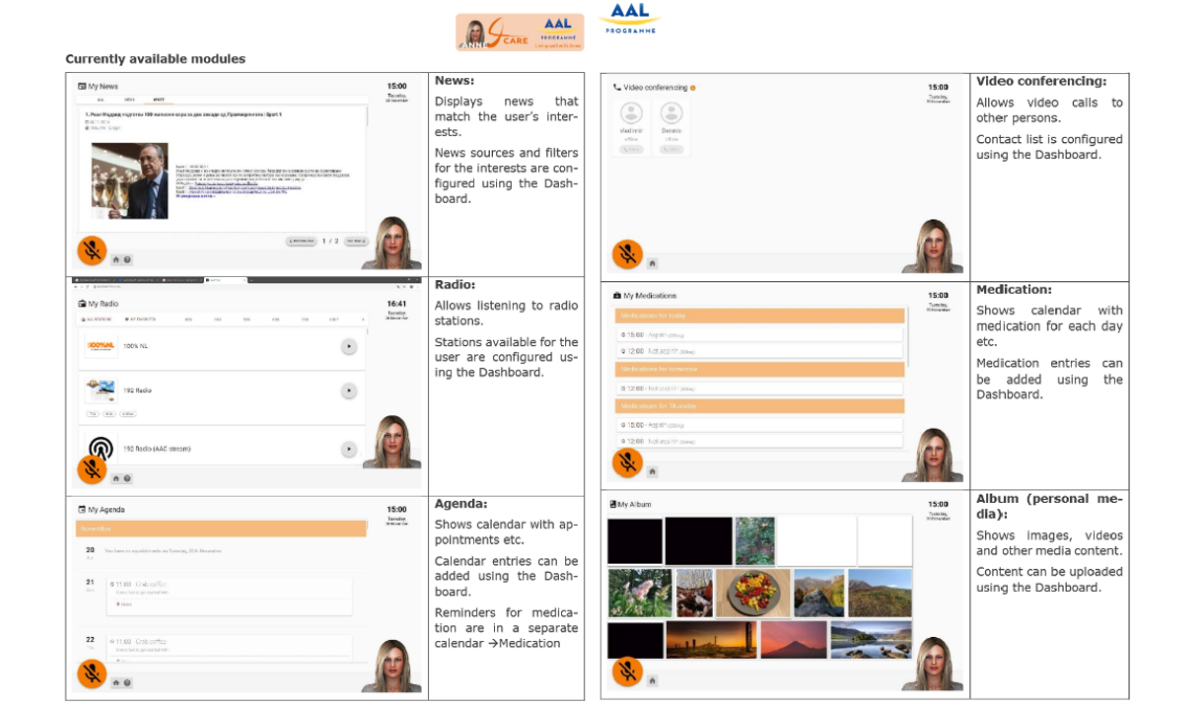
Screenshots of current available modules showing an example layout. The layout can be different, or the module hidden or disabled, depending on user abilities.

**Figure 2:**
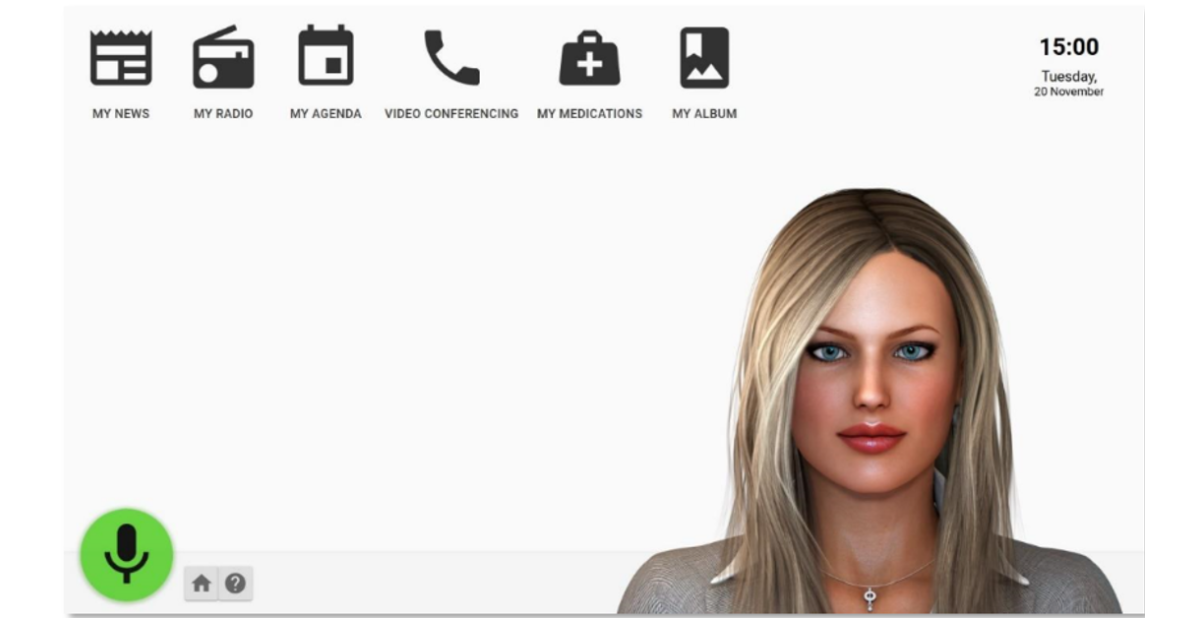
Home screenshots.

The virtual character works on a Surface Pro tablet with the Microsoft Windows 10 operating system. The following languages are currently available: Dutch, English, German, Italian, and French. Anne can support people with dementia in all aspects of daily life (Figures 1 and 2): communication with the outside world, keeping track of items on the personal calendar, daily structure, medication, reading the news, and relaxation (games and music). All these functionalities and features were developed following a user-driven approach [14], with the engagement of a multidisciplinary team and the involvement of users in the requirements definition process [15,16].

Users can interact with Anne through 2 channels: (1) a visual and haptic channel, via a Material–user interface graphical user interface (looking at the screen and touching the screen) and (2) an acoustical channel, via a voice user interface (listening to the avatar’s voice and speaking to the avatar). Anne’s voice user interface consists of automated speech recognition and text-to-speech functions. The user can always select which channel to use; that is, all commands must be accessible through touch and speech. During the requirements analysis, the clinical staff involved in the project suggested a suitable target group for the system—active and independent users able to interact with the avatar, discuss their thoughts, and express their opinions about Anne and who are competent and able to answer the protocol used for gathering data before and after the trial. Moreover, for safety reasons, individuals in the advanced stage of dementia were excluded from this study because dramatic swings in mood and behavior can be frequent, and it is not predictable how they can react in front of an unknown virtual assistant [16].

### Subjects

We enrolled 20 volunteers diagnosed with dementia in the study. Inclusion and exclusion criteria for enrollment have been presented in Textboxes 1 and 2.

In total, 14 family caregivers (eg, 9 spouses and 5 sons) were involved in the study.

This study was approved by the local ethics committee, and informed written consent was obtained from all subjects.

**Inclusion criteria for the study.**

**Inclusion Criteria**

- Age of 65 years or older
- Living independently
- Mini-Mental Status Examination [17] score between 24 and 27
- Ability to understand and sign the written informed consent

**Exclusion criteria for the study.**

**Exclusion Criteria**

- The presence of at least one of the following criteria excluded the user from enrollment:
  Lack of written informed consent
  Presence of an unstable chronic condition, with a Mini-Mental Status Examination score <24
  Presence of severe physical illness or disabilities that could be aggravated through the use of Anne

### Recruitment Procedure

The enrollment and recruitment strategy was implemented in the city of Ancona at the Neurology Unit of the National Institute of Health and Science on Aging (IRCCS INRCA [Istituto di Ricovero e Cura a Carattere Scientifico Istituto Nazionale di Riposo e Cura per Anziani]). According to the Living Well With Anne project activities, a staff composed of 2 psychologists identified 25 eligible participants among those who had regular access to the Alzheimer daily center. Each participant was invited to test the virtual agent, Anne. The 20 users who voluntarily participated in the study and used the system were enrolled. Their caregivers were also invited to participate in the research. Five family members refused to participate in the study because of a lack of time and effort in providing care.

### Study Design

The field trial ran for 4 weeks in participants’ homes. The entire study was managed by skilled personnel and researchers who ensured the supervision of tests and technical assistance during the period of interaction with the system. Each enrolled subject was introduced to Anne, received general training on its correct use, and returned home with a printed user manual with step-by-step instructions and a dedicated phone number to call in case of technical problems or doubts.

This study used a mixed methods approach, balancing quantitative and qualitative instruments to gather data from users.

Users responded to the following tests at the beginning and end of the 4 weeks of use:

1. Quality of life in older adults with cognitive impairment (Quality of Life in Alzheimer Disease scale [QOL-AD]) questionnaire [18,19]. The QOL-AD questionnaire has 13 items covering physical health, energy, mood, living situations, memory, family, marriage, friends, chores, fun, money, self, and life as a whole. The assessment is scored on a 4-point Likert scale ranging from 1 (poor) to 4 (excellent), with total scores ranging from 13 to 52.
2. The Almere model [20], which is a Likert scale–based questionnaire designed primarily to measure older adults’ acceptance of socially assistive robots. Almere measures the acceptance and attitudes of older adults through 12 constructs: (1) anxiety, (2) attitude toward technology (ATT), (3) facilitating conditions (FCs), (4) intention to use, (5) perceived adaptiveness (PAD), (6) perceived enjoyment (PENJ), (7) perceived ease of use (PEOU), (8) perceived sociability, (9) perceived usefulness (PU), (10) social influence, (11) social presence (SP), and (12) trust.

At the end of the period, users also responded to the questionnaires below:

1. The System Usability Scale (SUS) [21], a questionnaire that provides a quantitative measure of how usable a system is based on 10 statements rated by a 5-point Likert scale scored from 0-100, with 100 indicating perfect usability.
2. The closeness scale [22], which is a measure of self-other inclusion and relationship closeness. It was used to evaluate the closeness with the avatar at the end of the usage period.
3. Some unstructured short questions that were asked to users to record the general impression of the system (ie, role of Anne as virtual assistant, if Anne could have an impact on their well-being) and the major discomfort issues perceived during the use period.

Except for the closeness scale and unstructured short questions, family caregivers responded to the same scales. However, because of the overburden of caregivers, time-consuming qualitative data were not gathered from them.

Each instrument was verbally administered in a face-to-face session by a trained psychologist who entered the response on a paper version of each instrument.

During the 4 weeks of use, telemetry data were collected to track every event caused by an activity of the user on the tablet. Telemetry is the process of collecting data about remote objects and sending it to a computer electronically. These activities include clicks on the touch screen or voice interaction. Moreover, the used feature types such as games or medication reminders, were also recorded. All these activities were timestamped and therefore enabled a comprehensive analysis of the user’s behavior throughout time. Compared with surveys, this usage data is especially suited to detect problems and evaluate the status quo [23].

### Statistical Analysis

Continuous variables were reported as mean and SD, whereas categorical variables were expressed as absolute numbers and percentages. The Almere model [20] was used as the main instrument to acquire quantitative acceptance data. Negative questions were recoded (anxiety questions 1, 2, 3, and 4; PENJ question 20; PEOU questions 21, 24, and 25; and SP question 36). The questions and constructs of the Almere model are shown in Multimedia Appendix 1.

Changing of the acceptance level from pretest to posttest for both users and caregivers were reported as mean and SD of each item of the Almere model. Two-tailed paired samples *t* tests were conducted to compare the acceptability of Anne by older adults and caregivers throughout time (pretest and posttest). Closeness and perceived relations were reported as absolute numbers and percentages. Finally, usability was measured with the SUS [21], with item values reported as mean and SD.

## Results

### Overview

Recruitment began in October 2019, and the trial was initiated in December 2019. Enrollment was completed in January 2020. The sample of older adults comprised 20 users (mean age 75.5 years, SD 4.2), of whom 30% (6/20) were male and 70% (14/20) were female. A large percentage of participants were married (17/20, 85%) with a medium or high level of education. Only 6 participants had previous experience using tablets for leisure activities. The caregivers (mean age 66.4 years, SD 12.6) were proportionally male and female and had a medium or high level of education. The general quality of life was in between the fair and good perception and maintained this level during the 4 weeks of the study (Table 1).

**Table 1:** Older adults and caregivers characteristics.

Characteristics		Older adults (n=20)	Caregivers (n=14)
Age (years), mean (SD)		75.5 (4.2)	66.4 (12.6)
**Gender, n (%)**			
	Male	6 (30)	6 (43)
	Female	14 (70)	8 (57)
**Marital status, n (%)**			
	Married	17 (85)	11 (79)
	Full-time relationship	0 (0)	1 (7)
	Separated	0 (0)	0 (0)
	Divorced	1 (5)	0 (0)
	Single	1 (5)	2 (14)
	Widowed	1 (5)	0 (0)
**Education, n (%)**			
	No education	0 (0)	0 (0)
	Primary	7 (35)	2 (14)
	Secondary	4 (20)	5 (36)
	Tertiary	9 (45)	7 (50)
MMSE^a^, mean (SD)		25.2 (1.3)	N/A^b^
QOL-AD^c^ pretest, mean (SD)		28.5 (6.6)	35.5 (5.7)
QOL-AD posttest, mean (SD)		28.9 (7.8)	34 (7.6)
Delta QOL-AD, mean (SD)		0.4 (4.6)	−1.5 (3.8)

^a^MMSE: Mini-Mental Status Examination.

^b^N/A: not applicable.

^c^QOL-AD: Quality of Life in Alzheimer Disease scale.

### Acceptance

As reported in Multimedia Appendix 1, the results show that older adults became less anxious (anxiety, *P*=.007) during the 4 weeks of use. Positive changes among constructs were observed for PENJ (*P*=.04) and SP (*P*<.001). Other constructs such as ATT, PAD and FC did not change or nearly changed during the period of use. In contrast, PU (*P*=.02) and trust registered a negative change during the time of use.

For informal caregivers, the constructs of anxiety, FC, SP, and PEOU did not change or nearly changed during interactions with Anne. The constructs that registered negative changes were ATT (*P*=.15), PAD (*P*=.02), PENJ (*P*=.005), perceived sociability (*P*=.005), PU (*P*<.001), and trust (*P*=.01).

### Closeness Scale

**Figure 3:**
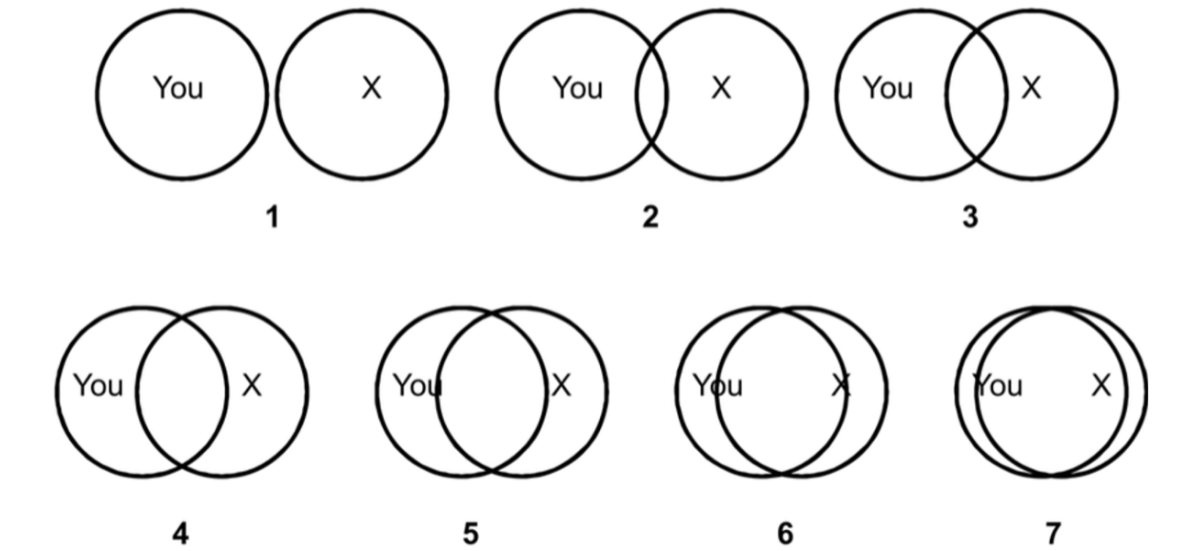
The seven pairs of increasingly overlapping circles describing the relationship between the older users and Anne.

The closeness scale aimed to assess the perceived relationship by asking respondents to evaluate their relationship with Anne. They had to select 1 of 7 pairs of increasingly overlapping circles that best described their relationship with Anne. In each pair of circles, one circle referred to the respondent and the other circle referred to Anne. A larger overlap indicated a closer relationship. For the analysis, visualization was numbered as follows: 1=no overlap, 2=little overlap, 3=some overlap, 4=equal overlap, 5=strong overlap, 6=very strong overlap, and 7=almost total overlap (Figure 3).

Older adults visualized their relationship with Anne with some overlaps (8/20, 42%) or no overlap (6/20, 26%). Minor percentages are attributed to strong, equal, or little overlap (2/20, 11%).

### Usability

All participants successfully completed the SUS. The SUS is scored out of 100, with a higher score indicating greater perceived usability. Anne received a mean score of 67.1 among older adults and 71.4 among caregivers. Both scores were compared and interpreted considering the acceptable average value of 68 (SD 12.5), which was determined for a variety of products and tools, including websites and technologies, provided by Sauro and Lewis [24] after the analysis of more than 5000 user scores encompassing almost 500 studies. As the virtual assistant used in this research was a system prototype and not a product ready for the market, it reached a positive result even if the score was below the acceptable average score of 68 in the case of seniors who were Anne’s primary users. From the analysis of the single items reported in Table 2, participants perceived Anne as easy to use and well integrated, thus instilling confidence during use and for the idea that people could quickly learn her major functionalities.

**Table 2:** System Usability Scale average scores among participants.

SUS^a^ items	Older adults, mean (SD)	Caregivers, mean (SD)
SUS-1. I think that I would like to use this system frequently	3.8 (1.3)	3.2 (1)
SUS-2. I found the system unnecessary complex	1.8 (1)	1.5 (0.9)
SUS-3. I thought the system was easy to use	4.1 (1.2)	4.2 (0.9)
SUS-4. I think that I would need the support of a technical person	2.9 (1.5)	2.2 (1.4)
SUS-5. I found the various functions well integrated	3.7 (0.9)	3.6 (0.9)
SUS-6. I thought there was too much inconsistency	2.4 (1.3)	2.1 (1.4)
SUS-7. I would imagine that most people would learn quickly	3.9 (0.9)	3.8 (0.7)
SUS-8. I found the system very cumbersome	2 (1.1)	1.6 (0.9)
SUS-9. I felt very confident using the system	3.3 (1.5)	3.2 (1)
SUS-10. I needed to learn a lot of things before I could get going	2.9 (1.3)	1.9 (0.9)
SUS score	67.1 (23.3)	71.4 (17.6)

^a^SUS: System Usability Scale.

### General Impression of the System and Major Discomforts

Some discomfort issues related to the speech command were pointed out in the unstructured short questions asked to users:

The speech command does not work well. Sometimes Anne answers other questions, and this is very frustrating, making me feel insecure. Maybe this is because of me as I have no tech skills.

About the news service, a participant mentioned the following:

Anne’s speech is poor, does not stop for punctuation when reading, always the same monotonous tone. Moreover, Anne only reads the title and it is not possible to listen to the full article.

Medication and gaming functions were the most successful services:

I often take it at the wrong time. I was very precise in my medicine intake thanks to Anna.

I think that having an assistant who reminds me is a great help.

The games help me keep my mind active.

I used to play memory games and puzzles every night. I think it is perfect to stimulate my memory.

Games helped me feel less lonely.

In addition, caregivers also really appreciated these functions:

I was glad to see my mother-in-law doing something new during the day.

The general impression was good among older users:

Anne kept me company when I was bored or alone.

I enjoy talking to Anna. Every day I say ‘good morning, Anne.’

When I was lonely, I used to talk to Anna.

Most older adults perceived Anne as a friend (6/14, 40%) or did not perceive any role to attribute (4/14, 30%). The remaining 30% (4/14) perceived the ECA as an assistant (2/14, 15%) and secretary (2/14, 15%).

When asked if Anne was seen as a way to improve their well-being, seniors responded positively (14/20, 70%), with a slight difference detected between male (4/6, 66%) and female (10/14, 71%) users. Among these users, the sense of well-being was related to the match with memory (6/17, 30%), ability to do things for fun (3/17, 15%), and mood (2/17, 10%). Female users mostly associated to the match with memory, whereas males identified a match with mood. The remaining 30% (6/17) did not find any connection between quality of life and Anne.

### Telemetry Data

Two distinct classes of events were established to meaningfully analyze telemetry data. Transition events describe events for navigating through Anne, which are mostly events caused by touching on the device’s touch screen, and target events that include using Anne’s actual features, such as reading the news and listening to the radio. Because of the similarity with the mouse navigation, a touch on the device’s touch screen is called a click in the sequel. Let us illustrate the target and transition events in a realistic example. A user would like to read a news article. Because he just started using Anne, he navigates by mistake to the game menu, realizes that and then navigates back to the main menu. Now, he navigates to the news menu and clicks on a news article that he intended to read. Each navigation caused a transition event as well as a click on the news article, resulting in 4 transition events and 1 target event. The number of transition events required to reach a target event depends on the target event itself. However, because of the user-friendliness of Anne, a target event usually requires only 1 to 2 transition events. Moreover, we see from the example how the distinction between transition and target events helps us assess the usability and learning effects of handling Anne.

During the observed trial period, 20 users actively performed 93,299 events. The most popular feature was games (52,008 events), followed by medication (2205 events), news, and radio (with 931 and 881 events, respectively). The high number of events for games is because of Anne’s design. A new event is created every time a user starts, quits, and restarts a game. These user actions are rather common in a game, and consequently, game events were very common. Nevertheless, as previously mentioned, games were a very popular feature.

From a telemetry perspective, the number of touch screen clicks before a target event can be seen as a measure of usability. Struggling users intuitively require many clicks because they try many different possibilities; hence, purposeful and quick handling is not possible. Furthermore, 80% (16/20) of the sample, that is, 16 users, only required one click on average for their target event. Thus, users seem to handle Anne very well.

Usability also has an impact on user behavior throughout time. At the beginning, a user using a new device is in an exploratory period, trying out the many features and not knowing how to handle the device very well. After some time, a user masters use and knows exactly how to handle the device. Hence, actions become very efficient and purposeful, and the efficiency in handling the device increases meaningful use. This user journey was also detected in the case of Anne.

**Figure 4:**
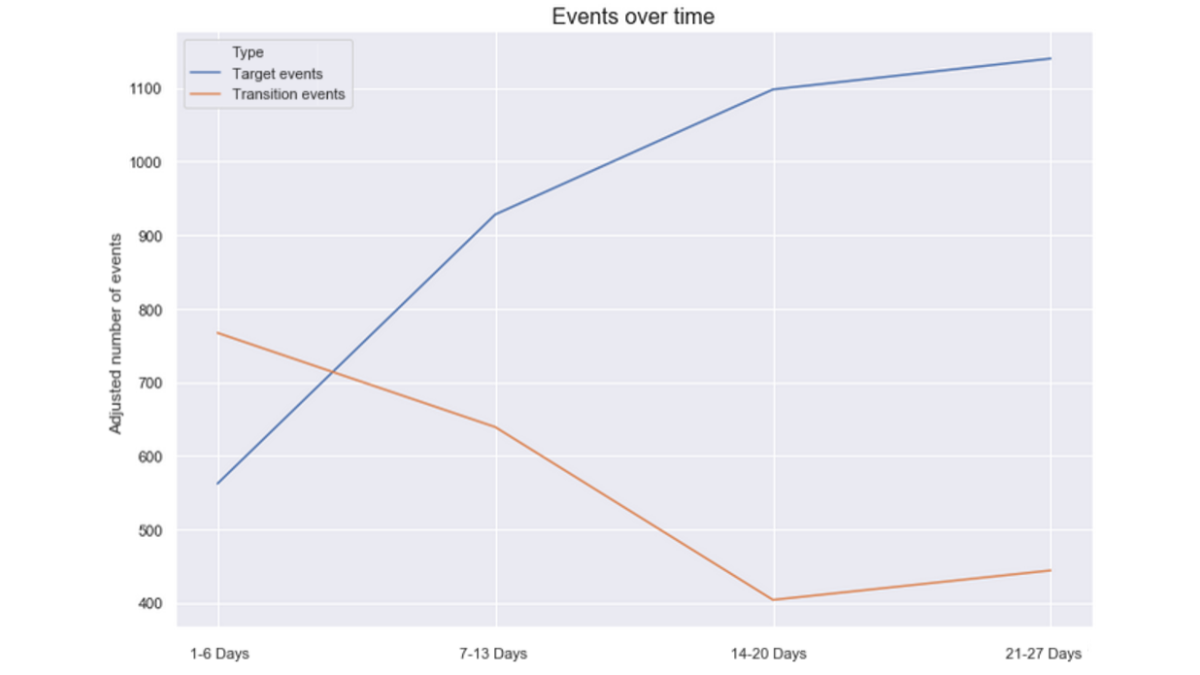
Target and transition events over time.

As previously defined, transition events mainly consist of touch screen clicks and on comments provided by Anne. Thus, a large number of transition events indicate problems with handling Anne, whereas a large number of target events show actual increased purposeful activity and interest. To capture user behavior, we decomposed the active field trial time range into 4 periods. The first period lasted from day 1 to day 6, the second from day 7 to day 13, the fourth from day 14 to day 20, and the final period from day 21 to day 27. We then analyzed the number of target and transition events during these periods. As the number of users varied in these periods, the number of target and transition events was adjusted according to the respective number of users. The corresponding visualization is shown in Figure 4.

In the first and second periods, the number of transition events was 767 and 639, respectively, whereas the number of target events was 562 and 928. In these periods, the number of transition events was comparatively high, whereas the number of target events was rather low. Consequently, user exploration took place during the first 2 periods; this contrasts with the 2 subsequent periods. In the third and fourth periods, the number of target events was 1098 and 1140, respectively, whereas the number of transition events was 404 and 444, respectively. Thus, we detected a sharp increase in target events and a decrease in transition events with time. Here, the users efficiently handled Anne and had many meaningful interactions, that is, target events.

Using similar measures, weekly reports were automatically generated to summarize the usage and detect potential technical problems of users with Anne. For example, if a user stopped using Anne because he was overwhelmed. Caregivers could respond to these problems with suitable assistance and thus prevent users from becoming frustrated with Anne. This was especially helpful for the typology of users involved in this study.

Voice interactions were also assessed. Compared with 853 successful voice interactions, users still preferred the touch screen in 55,442 cases to realize their purposes. Within voice interactions, users primarily preferred the feature news in 342 cases, followed by medication in 175 cases. Touchscreen interactions were dominantly used for game features, with 52,008 cases. For example, playing a puzzle or card game really requires touch screen interactions. In this context, voice interactions are inefficient and would be very unintuitive. As games were the most popular feature by far, this had a significant impact in favor of touch screen interactions. Another possible reason for the prevalence of touch screen interaction could be that users may not be natively familiar with such a new and innovative technology; in particular, if we consider the advanced age of users.

## Discussion

### Principal Findings

Overall, participants involved in this study demonstrated a positive approach to Anne: after 4 weeks of use, they were less anxious about interaction with Anne and more skilled in basic functionalities, and half of them perceived a role for the ECA. None of the participants withdrew from the trial, and they all provided useful feedback to facilitate the understanding of the data gathered. On the basis of these findings, it was found that older users were particularly engaged in providing significant responses and participated in improving the system. Some of them clearly discussed how technical problems related to speech recognition had a negative impact on intention to use, adaptiveness, usefulness, and trust. This impact emerged from the qualitative data reporting discomfort issues related to speech commands. The innovative aspect of Anne, as well as of other ECAs, is that users can interact with the system through voice and by stimulating a more human-like interaction rather than just navigating with the touch screen. Unfortunately, our study confirms that the quality of automatic speech recognition and synthesis is still a technical issue and has room for improvement [7-11], whereas touch screen modality is almost stable and positively used by people living with dementia [3,25,26]. The use of voice could also be relevant for older users in general and even more so for those who, for example, have motor skill impairments such as Parkinson disease. Moreover, it could also reduce the feeling of loneliness, which is a public health concern affecting our aging society globally [27].

Despite this issue, the closeness scale [22] analysis showed that Anne was perceived as a companion able to support memory and enjoyment needs. Anne served as a source of entertainment and as a way to handle adherence to medication plans. This is evident from telemetry data insights, which enabled the user-centric design analysis of Anne. For instance, it opens the idea for further developing actions to improve the game and medication features as well as setting new incentives for less-used features. Despite these results, the data have raised authors’ awareness to the fact that ECAs could be a promising way to cope with the health and well-being of people with dementia if they are designed, developed, and assessed around and with the users. The first challenge is to target the disease process from its earliest stages and follow the person throughout the journey to foster healthy aging and improve the lives of older people, their families, and the whole community.

Our study recruited 20 users in the early stages of dementia who scored between 24 and 27 in the Mini-Mental State Examination [17], as the main objectives were to assess users’ acceptance and the usability and feasibility of operating the system. In this stage, users used devices independently or, in the case of those with poor digital experience, they were still able to manage routine changes, such as introducing a new device into their daily life. In this study, users also benefited from general training on the correct use of the system, a printed user manual with step-by-step instructions, and a dedicated phone number to call for assistance in the case of technical problems or doubts. For people living with dementia, all novelties can become extremely distressing and disorientating. Therefore, becoming familiar with the technology at an early stage is fundamental, as it provides users with continuous support.

It is well known that the abilities and needs of people with dementia and those who provide care for them change along the path defined by the progression of symptoms, as does the ability to cope with them. These changing needs also mean that some technologies will be more appropriate or effective at different stages of dementia [28]. Most interventions targeting people in the early stages of dementia and their caregivers aim to support people’s memory and their ability to live independently. Examples of technologies falling into this category are GPS, communication devices, and other technologies that can help mitigate memory problems (eg, medication reminders, locators, voice cues to help perform daily activities, and *dementia-friendly* versions of household gadgets) and tools promoting the self-management of health [29]. For people within moderate to severe stages of dementia (eg, with an Mini-Mental State Examination score between 20 and 9), the largest set of technologies are those that enhance safety (ie, fall detectors, motion-sensitive lights, sensors measuring room temperature and raising alarms when it gets too warm or too cold, and cooker or smoke alarms). At this stage of dementia, active use of safety technology tends to shift to the carer, whereas people with dementia often become less active users [28]. Moreover, examining feedback from the point of view of family caregivers, as in our study, or other caregivers (ie, nurses, health care professionals, or care workers who work with people with dementia), could build more awareness on how to develop effective technologies.

We cannot achieve the challenge of targeting the disease process from its earliest stages without changing the way of thinking, feeling, and acting toward people living with dementia and their complex needs. Thus, the second challenge is to support people living with dementia in doing what they need and decide, recognizing their purpose, identity, and independence. The key point is to avoid any type of stereotype about the experience of living with dementia and the opportunities that ECAs could offer to really address individuals’ medical, cognitive, psychological, environmental, cultural, and social needs [30]. According to the World Health Organization global strategy on aging and health [31-33], such action could value the person’s functions and needs. This means that technologies must continue to monitor health and safety as primary needs. However, it is necessary to support and maintain physical and mental capacity throughout the life course by providing opportunities for leisure and social activities to facilitate inclusion and participation, thus reducing loneliness and social isolation [34]. Supporting higher-level needs such as belonging, self-esteem, identity, and self-actualization [35] is the aim of the next generation of technologies.

Older adults are bearers of value for the design of technologies and beneficiaries of such systems. If the meaningful engagement of patients with dementia is essential for setting the future of ECAs, the two challenges depend on how much researchers, medical scientists, technology developers, and social and business innovators are ready to agree on a common vision concerning healthy aging as the way of developing and maintaining functional ability enabling well-being in old age [34-36]. In this study, the connectivity between information and communication technologies sectors, clinical staff, and research fields was ensured by adopting a user-centric design approach [14], enabling Anne’s original version to be adapted to the specificities of users. Design for older adults is typically a multidimensional process involving significant time and cost in thinking, problem-solving research, iterative testing, and redesigning to meet the needs, capabilities, and limitations of users [37]. However, there are still great opportunities to be discussed and learned from the untold stories of implementing a user-centered design to create more efficient, effective, and sustainable eHealth solutions [38]. The COVID-19 pandemic highlights the significant value of digital technologies for reaching older adults, especially if frail and with multiple chronic diseases. In times of social distancing and reduced access to health services, a wide scope for innovations is covering clinical and cultural difficulties caused by the coronavirus pandemic and opening the great opportunity to increase the quality of services and access to health information.

### Comparison With Previous Works and Limitations

Longer trials are needed to measure changes in user experience and familiarity with the system. This research focused on 4 weeks of interaction between 20 seniors with dementia and the agent Anne in a home setting. This framework is rare in ECA research, which mainly comprises studies on short-term interaction in a controlled environment [13] and smaller sample size enrollment [7-12]. To the best of our knowledge, this is one of the few studies on the use of ECA among people living with dementia and their caregivers. Despite these strengths, 4 weeks were not enough to evaluate a significant level of acceptability and usability, even in light of the technical discomfort related to automatic speech recognition. Moreover, the specific Italian national context and culture could be seen as a bias and a significant limitation that does not allow for the generalization of results. Nevertheless, conducting methodologically sound scientific research in dementia care and support community is an urgent step forward [39,40]. Specifically, the possibility of running randomized control trials, enrolling a larger sample, and gathering data before and after longer interventions through robust methods remains a key challenge for the whole sector of innovation technologies [41-43]. Another key step could be to further analyze the user characteristics that match the positive acceptance of such systems and consent to better profile future customers. From a technical point of view, this study mapped how the readiness level of ECA-based interventions grew across the years, shifting from a display on standard television sets [7] and computer screens [8-12] to mobile stand-alone solutions such as in the specific case of Anne. At first, ECA functions matched the physiological, comfort, and attachment needs [7,8,12] sought, mainly to overcome memory problems, guide patients in their daily activities, and meet their need for communication and social interaction. Advancements then started to propose features such as guiding through a task, cognitive stimulation exercises, and attention management [9,10]. In comparison with these predecessors, Anne represents a step forward by offering a multipurpose tool integrating features such as reminders (personal and medication agenda), communication (video calls), information (news), and entertainment (games and music) that support users in all aspects of daily life.

### Conclusions

This study aimed to evaluate the usability and acceptance of the ECA Anne by people living with dementia. It demonstrated the ability of target users to use the system independently in their home environment and receive valuable information from it. Overall, participants shared good engagement with the system, approaching the virtual agents as a companion able to support memory and enjoyment needs. Therefore, this study provides evidence for using ECAs as future eHealth systems to address the basic and higher-level needs of people living with dementia. This specific field of research is novel and poorly discussed in the scientific community. This could be because of its novelty, yet there is an urgent need to strengthen data, research, and innovation to accelerate the implementation of ECAs as a future way of offering nonpharmacological support to community-dwelling people with dementia. In our vision, this primarily means collaboration among interdisciplinary research networks, medical scientists, technology developers, and social and business innovators and the direct engagement of older adults and their formal and informal caregivers. Furthermore, sharing the strengths and weaknesses of research is fundamental for building common knowledge from previous studies. In the midst of the COVID-19 pandemic, these key points could prove significant in improving health care services. eHealth technologies have considerable challenges to overcome, but the opportunity to increase the quality of services and access to health information for users can really make a difference in these times of the pandemic.

## Appendix

Multimedia Appendix 1 Almere model constructs and items.

## Abbreviations

ATT: attitude toward technology
ECA: embodied conversational agent
FC: facilitating condition
IRCCS INRCA: Istituto di Ricovero e Cura a Carattere Scientifico Istituto Nazionale di Riposo e Cura per Anziani
PAD: perceived adaptiveness
PENJ: perceived enjoyment
PEOU: perceived ease of use
PU: perceived usefulness
QOL-AD: Quality of Life in Alzheimer Disease scale
SP: social presence
SUS: System Usability Scale
