# Supplementary material for: Usability and Acceptance of the Embodied Conversational Agent Anne by People With Dementia and Their Caregivers: Exploratory Study in Home Environment Settings
Source: JMIR Mhealth Uhealth. 2021 Jun 19;9(6):e25891. doi: 10.2196/25891 (PMC8386369; doi:10.2196/25891)
Supplement: Multimedia Appendix 1 [file mhealth_v9i6e25891_app1.docx]

### **Multimedia Appendix 1**

Almere Model constructs and items among users and caregivers (mean±sd)

| **Almere Model Items** | **Older adults** | | | **Caregivers** | | |
| --- | --- | --- | --- | --- | --- | --- |
|  | **pre** | **post** | **p** | **pre** | **post** | **p** |
| **Anxiety (ANX)** | 3.97±0.66 | 4.62±0.75 | **.007** | 4.82±0.31 | 4.82±0.37 | 1.000 |
| 1 When using Anne, I am afraid to make mistakes with it | 4.00±0.85 | 4.65±0.74 | **.015** | 4.78±0.42 | 4.78±0.42 | 1.000 |
| 2 When using Anne, I am afraid to break something | 3.80±0.89 | 4.65±0.74 | **.002** | 4.78±0.42 | 4.78±0.42 | 1.000 |
| 3 I find Anne scary | 4.10±0.64 | 4.65±0.74 | **.017** | 4.85±0.36 | 4.85±0.36 | 1.000 |
| 4 I find Anne intimidating | 4.00±0.79 | 4.55±0.94 | .061 | 4.85±0.36 | 4.85±0.36 | 1.000 |
| **Attitude (ATT)** | 3.61±0.49 | 3.65±0.84 | .857 | 3.95±0.38 | 3.47±0.85 | **.015** |
| 5 It is a good idea to use Anne | 3.95±0.60 | 3.80±1.00 | .481 | 4.14±0.53 | 3.78±0.97 | .096 |
| 6 Anne makes life more interesting | 3.05±0.68 | 3.30±0.97 | .330 | 3.71±0.72 | 3.07±1.07 | **.022** |
| 7 It’s good to make use of Anne | 3.85±0.67 | 3.85±0.81 | 1.000 | 4.00±0.39 | 3.57±0.85 | **.028** |
| **Facilitating conditions (FC)** | 3.85±0.51 | 3.65±1.00 | .408 | 4.14±0.41 | 3.92±0.43 | .082 |
| 8 I have everything I need to use Anne | 4.00±0.56 | 3.70±0.97 | .249 | 4.07±0.47 | 4.00±0.39 | .583 |
| 9 I know enough of Anne to make good use of it | 3.70±0.57 | 3.60±1.04 | .681 | 4.21±0.42 | 3.85±0.53 | **.019** |
| **Intention to Use (ITU)** | 4.05±0.70 | 3.36±1.17 | **.017** | 3.73±0.62 | 2.97±0.77 | **.006** |
| 10 I think I will use Anne during the next few days | 4.15±0.67 | 3.45±1.14 | **.015** | 3.92±0.47 | 3.07±0.73 | **.000** |
| 11 I’m certain to use Anne during the next few days | 4.00±0.72 | 3.35±1.18 | **.024** | 3.64±0.74 | 2.92±0.82 | **.019** |
| 12 I plan to use Anne during the next few days | 4.00±0.79 | 3.30±1.26 | **.023** | 3.64±0.74 | 2.92±0.82 | **.019** |
| **Perceived adaptiveness (PAD)** | 3.78±0.43 | 3.40±0.87 | .095 | 3.71±0.43 | 3.16±0.58 | **.015** |
| 13 Anne is adapted to my needs | 3.75±0.71 | 3.40±1.04 | .201 | 3.64±0.63 | 3.00±0.78 | **.022** |
| 14 Anne does what I need her to do at any particular moment | 3.70±0.57 | 3.20±1.00 | .066 | 3.50±0.65 | 3.28±0.72 | .459 |
| 15 Anne helps me when I consider it to be necesarry | 3.90±0.44 | 3.60±0.82 | .186 | 4.00±0.55 | 3.21±0.69 | **.015** |
| **Perceived Enjoyment (PENJI)** | 3.47±0.23 | 3.86±0.86 | **.041** | 4.15±0.19 | 3.50±0.71 | **.005** |
| 16 I enjoy talking to Anne | 3.95±0.51 | 3.65±0.98 | .186 | 4.00±0.39 | 3.21±0.69 | **.006** |
| 17 I enjoy doing things with Anne | 3.90±0.64 | 3.65±0.93 | .234 | 4.00±0.39 | 3.00±0.78 | **.003** |
| 18 I find Anne enjoyable | 4.00±0.32 | 3.90±0.91 | .606 | 4.14±0.36 | 3.64±0.74 | **.029** |
| 19 I find Anne fascinating | 4.00±0.45 | 3.80±0.89 | .359 | 3.85±0.86 | 3.57±0.64 | .365 |
| 20 I find Anne boring | 4.50±0.60 | 1.70±1.03 | **.000** | 4.78±0.57 | 4.07±1.07 | **.045** |
| **Perceived Ease of Use (PEOU)** | 3.28±0.66 | 3.61±0.96 | .129 | 4.10±0.75 | 4.21±0.78 | .603 |
| 21 I find it difficult to know how to use Anne | 3.90±0.78 | 4.00±1.25 | .748 | 4.42±0.93 | 4.14±1.02 | .453 |
| 22 I find Anne easy to use | 3.80±0.61 | 3.85±0.98 | .834 | 4.28±0.61 | 4.28±0.61 | 1.000 |
| 23 I can use Anne without any help | 3.40±0.88 | 3.55±1.35 | .685 | 4.28±0.72 | 4.14±1.09 | .671 |
| 24 I can only use Anne when there is someone around to help me | 2.45±1.05 | 3.05±1.39 | **.049** | 3.71±1.13 | 4.00±1.24 | .263 |
| 25 I can only use Anne when I have a good manual | 2.85±1.13 | 3.60±1.31 | **.018** | 3.78±1.12 | 4.50±0.51 | **.006** |
| **Perceived Sociability (PS)** | 3.66±0.39 | 3.35±0.74 | .075 | 3.91±0.43 | 3.19±0.68 | **.005** |
| 26 Anne is a pleasant conversational partner | 3.60±0.59 | 3.25±0.78 | .110 | 3.85±0.66 | 3.07±0.82 | **.003** |
| 27 I find Anne pleasant to interact with | 3.70±0.47 | 3.35±0.74 | .069 | 4.00±0.39 | 3.21±0.69 | **.003** |
| 28 I feel Anne understands me | 3.40±0.75 | 3.15±1.03 | .309 | 3.71±0.82 | 2.78±0.80 | **.017** |
| 29 I find Anne nice | 3.95±0.39 | 3.65±0.81 | .137 | 4.07±0.26 | 3.71±0.72 | .096 |
| **Perceived Usefulness (PU)** | 3.81±0.41 | 3.41±0.80 | **.023** | 3.92±0.41 | 2.66±0.64 | **.000** |
| 30 I find Anne useful to me | 3.75±0.55 | 3.30±0.97 | **.025** | 3.85±0.53 | 2.92±0.91 | **.002** |
| 31 It is convenient for me to have Anne | 3.90±0.30 | 3.60±0.68 | **.030** | 4.00±0.39 | 3.35±0.84 | **.022** |
| 32 Anne can help me with many things | 3.80±0.52 | 3.35±0.93 | **.046** | 3.92±0.47 | 3.07±0.82 | **.005** |
| **Social Presence (SP)** | 1.51±0.44 | 2.34±0.51 | **.000** | 1.58±0.57 | 1.42±0.77 | .603 |
| 33 When interacting with Anne I felt like was talking to a real person | 1.60±0.82 | 2.10±1.07 | .106 | 1.50±0.75 | 1.28±0.61 | .487 |
| 34 It sometimes feels as if Anne is really looking at me | 1.60±0.75 | 2.35±1.22 | **.032** | 1.78±1.31 | 1.42±1.08 | .496 |
| 35 I imagine Anne to be a living creature | 1.20±0.41 | 1.55±0.94 | .185 | 1.42±0.64 | 1.28±0.82 | .635 |
| 36 I often think Anne is not a real person | 1.65±0.93 | 1.60±0.82 | .863 | 1.71±1.06 | 1.50±1.09 | .609 |
| 37 Sometimes Anne seems to have real feelings | 1.50±0.76 | 1.30±0.47 | .297 | 1.50±1.09 | 1.50±1.28 | 1.000 |
| **Trust (TR)** | 3.75±0.50 | 3.42±0.89 | .103 | 3.71±0.46 | 3.03±0.81 | **.012** |
| 38 I trust Anne if she gives me advice | 3.75±0.55 | 3.40±0.94 | .110 | 3.64±0.49 | 3.00±0.87 | **.022** |
| 39 I follow the advice Anne gives me | 3.75±0.55 | 3.45±0.88 | .137 | 3.78±0.57 | 3.07±0.82 | **.012** |
